# Differential metabolic responses of mouse Leydig and spermatogonia cells to radiofrequency electromagnetic field exposure

**DOI:** 10.3389/fpubh.2025.1623701

**Published:** 2025-09-19

**Authors:** Xia Miao, Yanyun Lin, Juan Guo, Jiajin Lin, Peng Gao, Wei Zhang, Lihua Zeng, Guozhen Guo, Jing Li

**Affiliations:** ^1^Department of Radiation Medical Protection, School of Military Preventive Medicine, Air Force Medical University, Xi'an, China; ^2^The Ministry-of-Education’s Key Laboratory of Hazard Assessment and Control in Special Operational Environment, Air Force Medical University, Xi'an, China

**Keywords:** RF-EMFs, reproductive health, metabolomics, sensitivity, KEGG

## Abstract

**Introduction:**

Although existing studies have shown that radiofrequency electromagnetic fields (RF-EMFs) have a variety of effects on living organisms, the specific impact of RF-EMFs on the metabolism of reproductive cells and their underlying mechanisms remain unclear.This study aims to explore the effects of RF-EMFs on the metabolism of mouse Leydig cells (TM3) and spermatogonia cells (GC-1) through metabolomics analysis, revealing the potential mechanisms by which RF-EMFs affect reproductive health.

**Methods:**

We employed liquid chromatography-mass spectrometry (LC-MS) to analyze the metabolomic profiles of TM3 and GC-1 cells under two irradiation modalities: continuous and intermittent RF-EMF exposure. The data were further analyzed using KEGG pathway analysis to identify significantly enriched metabolic pathways. The ELISA (Enzyme-Linked Immunosorbent Assay) was used to detect glutathione levels.

**Results:**

Our results showed that continuous irradiation had a more pronounced impact on the metabolism of TM3 cells, primarily affecting amino acid metabolism, the citric acid cycle, ABC transporters, bile secretion, and glutathione metabolism. In contrast, intermittent irradiation mainly altered the levels of fatty acyls and purine nucleosides, with significant enrichment in purine metabolism, biosynthesis of unsaturated fatty acids, and fatty acid metabolism. Compared to TM3 cells, GC-1 cells exhibited lower sensitivity to RF-EMF irradiation. Both irradiation modalities affected purine metabolism and lysine degradation pathways in TM3 cells, suggesting that changes in ADP levels may serve as a key metabolic signature in the cellular response to RF-EMF exposure.

**Conclusion:**

Continuous irradiation significantly impacts TM3 cell metabolism, particularly amino acid and glutathione pathways, while intermittent irradiation mainly affects fatty acyls and purine metabolism. GC-1 cells show lower sensitivity to RF-EMF. ADP level changes may be a key metabolic signature of RF-EMF exposure.

## Introduction

The proliferation of wireless communication technologies, including mobile phones and Wi-Fi networks, has led to a dramatic increase in human exposure to radiofrequency electromagnetic fields (RF-EMFs). This has sparked significant concern regarding the potential health risks, particularly on reproductive health, given the sensitivity of reproductive cells to environmental stressors (1–5).

Some studies showed the effects of RF-EMFs on male reproductive health, revealing disruptions in testicular function, such as increased permeability of the blood-testis barrier (6–8), decreased sperm quality (9), impaired sperm DNA integrity (10, 11), inhibits testosterone secretion (12, 13) and influence the immunity and induce oncogenic risks in testicular (14). Additionally, RF-EMF exposure has been linked to oxidative stress in reproductive organs, characterized by elevated reactive oxygen species (ROS) levels and reduced antioxidant enzyme activity (15, 16). However, International bodies such as the World Health Organization (WHO) and the U. S. National Cancer Institute state that radiofrequency exposures below existing limits have not been shown to cause consistent adverse health effects. A WHO-coordinated systematic review of experimental studies in non-human mammals and human sperm *in vitro* concluded that RF-EMF exposure may adversely affect pregnancy rate and sperm count, whereas litter size was (17). Thus, the effect of radiofrequency exposures remains inconclusive and controversial. A growing body of evidence demonstrates that the frequency of RF-EMF markedly influences its biological impact on reproductive cells. For example, studies on Leydig cells have shown that exposure at 2,450 MHz increases ROS levels without compromising cell viability, whereas identical conditions at 1,800 MHz neither elevate ROS nor elicit any other detectable adverse effects (18). However, the biological effects and underlying regulatory mechanisms of 1,950 MHz RF-EMF—a mainstream carrier used in 3G uplink and 4 G LTE auxiliary bands—on reproductive cells remain largely undefined and require further investigation.

Metabolomics, a rapidly evolving field in bioanalytical chemistry, offers a powerful approach to investigate metabolic alterations in biological systems induced by external stimuli (19). By quantifying and annotating metabolites, metabolomics enables the identification of perturbed metabolic pathways, proteins, and genes, providing a comprehensive view of cellular responses to environmental factors (20).

In this study, we aimed to explore the metabolic effects of RF-EMF irradiation on mouse Leydig cells (TM3) and spermatogonia cells (GC-1) using Liquid Chromatography–Mass Spectrometry-based (LC–MS-based) metabolomics. We compared the metabolic alterations induced by continuous and intermittent irradiation to elucidate the potential mechanisms by which RF-EMFs affect reproductive cell function. We hypothesized that different irradiation modalities would result in distinct metabolic signatures and different cells shows differential sensitivity to irradiation, offering new insights into the impact of RF-EMFs on reproductive health.

## Materials and methods

### Chemicals and reagents

LC-grade methanol, acetonitrile, ammonium acetate, and formic acid were sourced from CNW Technologies GmbH (CAS numbers: 67-56-1, 75-05-8, 631-61-8, 64-18-6 respectively, purity ≥ 99.9%, Shanghai, China). 2-Chlorophenylalanine-L was obtained from Shanghai PureChem Biotechnology Co., Ltd. (CAS number: 103616–89-3, purity ≥ 98%, Shanghai, China). High-purity deionized water was generated using a Milli-Q Integral water purification system (Merck Millipore, Darmstadt, Germany).

### Cell culture and ethics statement

The TM3 (mouse Leydig cell line) and GC-1 cell line (the mouse spermatogonia cell line) utilized in our experiments were acquired from the Shanghai Cell Bank of the Chinese Academy of Sciences. These cells were cultured in 10 cm Petri dishes coated with gelatin. The growth medium was a combination of Dulbecco’s Modified Eagle’s Medium DMEM F-12 (1: 1 ratio; sourced from Invitrogen, Carlsbad, United States), supplemented with 10% fetal bovine serum (FBS; Gibco, Grand Island, United States). Once the cells attained approximately 80% confluence, they were detached using 0.25% trypsin (Invitrogen) and subsequently employed for our experimental procedures. The cells were consistently maintained in an incubator at 37 °C, under humidified conditions with 5% CO_2_.

### RF-EMR exposure equipment and exposure parameter

The Xc-ELF irradiation system (sXc-1950, Zurich, Switzerland) was used for RF-EMF exposure. The system consisted of an RF generator, an arbitrary function generator, a narrowband amplifier, and rectangular waveguides. The specific absorption rate (SAR) was maintained at 3 W/kg, with temperature and CO_2_ levels controlled at 37 °C and 5%, respectively. The irradiation parameter of 3 W/kg falls between the occupational exposure limit (whole body: 4 W/kg, 6-min average) and the public exposure limit (whole body: 0.08 W/kg, 30-min average; head and trunk: 2 W/kg, 6-min average; extremities: 4 W/kg, 6-min average). The corresponding whole-body average SAR exceeds the public exposure limit but does not reach the occupational exposure limit. For continuous irradiation, cells were exposed to 1950 MHz GSM signals for 24 h. Intermittent irradiation involved 1-h exposure followed by a 1-h break, repeated over 24 h. Control groups were sham-exposed under identical conditions.

### Metabolite extraction

After being subjected to various radiation treatments for 24 h, the cells were harvested. Subsequently, extract solvent (acetonitrile-methanol–water, 2:2:1 ratio, containing an internal standard) was added to the samples. The samples were vortexed for 30 s, homogenized at 45 Hz for 4 min, and sonicated in an ice-water bath for 5 min. The homogenate and sonicate circle were repeated for 3 times, followed by incubation at −20 °C for 1 h and centrifugation at 12000 rpm and 4 °C for 15 min. The supernatants were transferred into LC–MS vials for analysis. The quality control (QC) sample was prepared by mixing an equal aliquot of the supernatants from all of the samples.

### LC-MS/MS analysis

LC-MS/MS analyses were performed using an UHPLC system (1,290, Agilent Technologies) with a UPLC HSS T3 column (2.1 mm × 100 mm, 1.8 μm) coupled to Q Exactive (Orbitrap MS, Thermo). The mobile phase A was 0.1% formic acid in water for positive, and 5 mmol/L ammonium acetate in water for negative, and the mobile phase B was acetonitrile. The QE mass spectrometer was used for its ability to acquire MS/MS spectra on an information-dependent basis (IDA) during an LC/MS experiment. MS raw data (.raw) files were converted to the mzML format using ProteoWizard, and processed by R package XCMS (version 3.2), including retention time alignment, peak detection and peak matching. Then the data were filtered and normalized to an internal standard for each sample was done subsequently. Next, missing values were replaced by the half of the minimum value found in the dataset by default. The preprocessing results generated a data matrix that consisted of the retention time (RT), mass-to-charge ratio (m/z) values, and peak intensity. OSI-SMMS (version 1.0, Dalian Chem Data Solution Information Technology Co. Ltd.) was used for peak annotation after data processing with in-house MS/MS database.

### Data analysis

Unsupervised principal component analysis (PCA) was utilized to display the separation of origin data. Partial least square discriminant analysis (PLS-DA) was implemented to obtain maximal covariance between variables and sample category in both positive and negative models. Variable Importance in Projection (VIP) value was obtained from each variable in the PLS-DA model. The Variables with VIP > 1 was further analyzed by independent samples two-tail Student’s *t*-tests to evaluate the significance of each variable. Fold change (FC) was calculated using average relative peak intensities in continuous irradiation group/control group, intermittent irradiation group/control group. Variables with VIP > 1.0, fold change > 1.2 or < 0.83 and *p*-value < 0.05 were defined as differential metabolites. The KEGG^1^ was explored to enrich metabolic pathways. OmicsNet website^2^ was applied to integrate networks from differential metabolites and regulatory target genes.

### ELISA measurement of glutathione (GSH) concentration in TM3 cell

The TM3 cells were exposed to continuous RF-EMR for a duration of 24 h. Following this continuous exposure, the concentration of GSH in the TM3 cells was measured using an Enzyme-Linked Immunosorbent Assay (ELISA) kit specific for GSH (Glutathione ELISA Kit, manufactured by elabscience, product code E-EL-0026).

### Statistical analysis

All data were presented as mean ± SEM and were analyzed by GraphPad Prism 7.04 software. Statistical evaluations were conducted using a two-tailed Student’s *t*-test, with significance set at *p* < 0.05. The comparisons involving more than two groups were analyzed by one-way ANOVA followed by Tukey’s HSD *post hoc* test. In the graphical representations, asterisks (*) indicate a statistically significant difference between two groups, specifically *** for *p* < 0.001.

## Results

### Metabolic profiling of TM3 cells after RF-EMF irradiation

**Figure 1:**
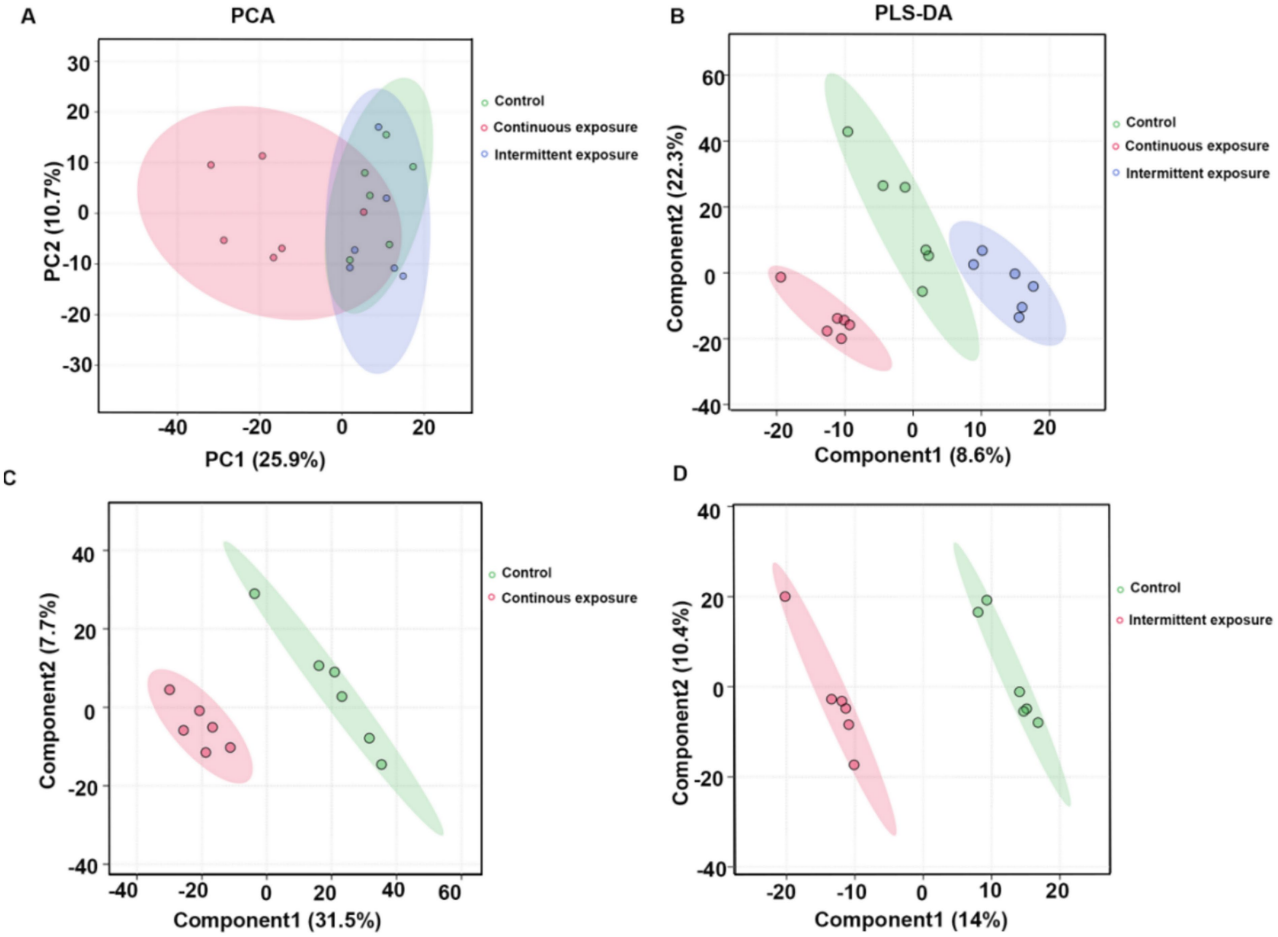
Metabolite profiling in TM3 cells after RF-EMF irradiation. **(A)** PCA score plots of different samples. **(B)** Partial least squares discriminant analysis (PLS-DA) scores of the metabolites in different groups. **(C)** PLS-DA scores of the metabolites in control and continuous exposure group. **(D)** PLS-DA scores of the metabolites in control and intermittent exposure group.

To investigate the metabolic effects of RF-EMF irradiation on TM3 cells, we performed non-targeted metabolomic analysis using liquid chromatography-mass spectrometry (LC-MS). Principal component analysis (PCA) was initially conducted to visualize the intrinsic metabolic differences between the control, continuous irradiation, and intermittent irradiation groups. The PCA score plots revealed distinct separation between the continuous irradiation group and the control group, indicating significant metabolic alterations induced by continuous RF-EMF exposure (Figure 1A). In contrast, the unsupervised PCA model did not clearly distinguish the intermittent irradiation group from the control, suggesting that continuous irradiation had a more pronounced impact on TM3 cell metabolism. The PLS-DA score plots demonstrated clear separation between the continuous irradiation group and the control group, with R2Y = 0.99 and Q2Y = 0.608 in positive ion mode, and R2Y = 0.987 and Q2Y = 0.563 in negative ion mode (Figure 1B). Similarly, the intermittent irradiation group showed good separation from the control group, with R2Y = 0.996 and Q2Y = 0.469 in positive ion mode, and R2Y = 0.99 and Q2Y = 0.676 in negative ion mode (Figures 1C,D). These results confirmed the reliability and predictability of the PLS-DA model.

### Metabolic profiling of GC-1 cells after RF-EMF irradiation

**Figure 2:**
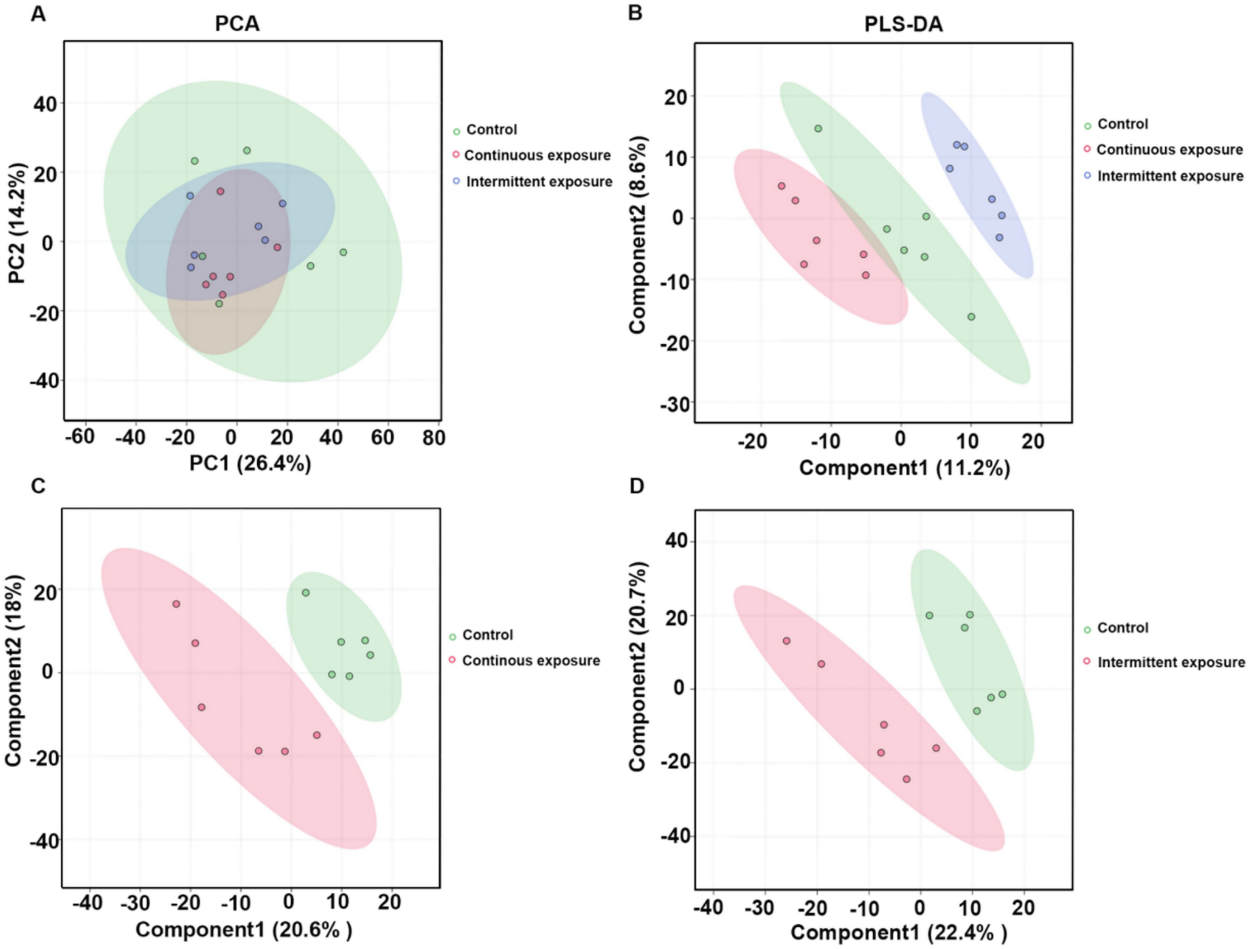
Metabolite profiling in GC-1 cells after RF-EMF irradiation. **(A)** PCA score plots of different samples. **(B)** Partial least squares discriminant analysis (PLS-DA) scores of the metabolites in different groups. **(C)** PLS-DA scores of the metabolites in control and continuous exposure group. **(D)** PLS-DA scores of the metabolites in control and intermittent exposure group.

We also conducted non-targeted metabolomic analysis in GC-1 cells, comparing the control group, continuous irradiation group, and intermittent irradiation group. The PCA score plots did not show clear separation between the groups (Figure 2A), suggesting that GC-1 cells exhibited lower sensitivity to RF-EMF exposure compared to TM3 cells. To further explore potential metabolic variations, partial least squares discriminant analysis (PLS-DA) was performed. The PLS-DA score plots demonstrated moderate separation between the continuous irradiation group and the control group, with R2Y = 0.993 and Q2Y = 0.52 in positive ion mode, and R2Y = 0.995 and Q2Y = 0.205 in negative ion mode (Figure 2B). Similarly, the intermittent irradiation group showed separation from the control group, with R2Y = 0.832 and Q2Y = 0.633 in positive ion mode, and R2Y = 0.763 and Q2Y = 0.349 in negative ion mode (Figures 2C,D). Although the separation was less pronounced than in TM3 cells, these results suggested that subtle metabolic changes in GC-1 cells after RF-EMF irradiation.

### Differential metabolite expression in TM3 and GC-1 cells after RF-EMF exposures

**Figure 3:**
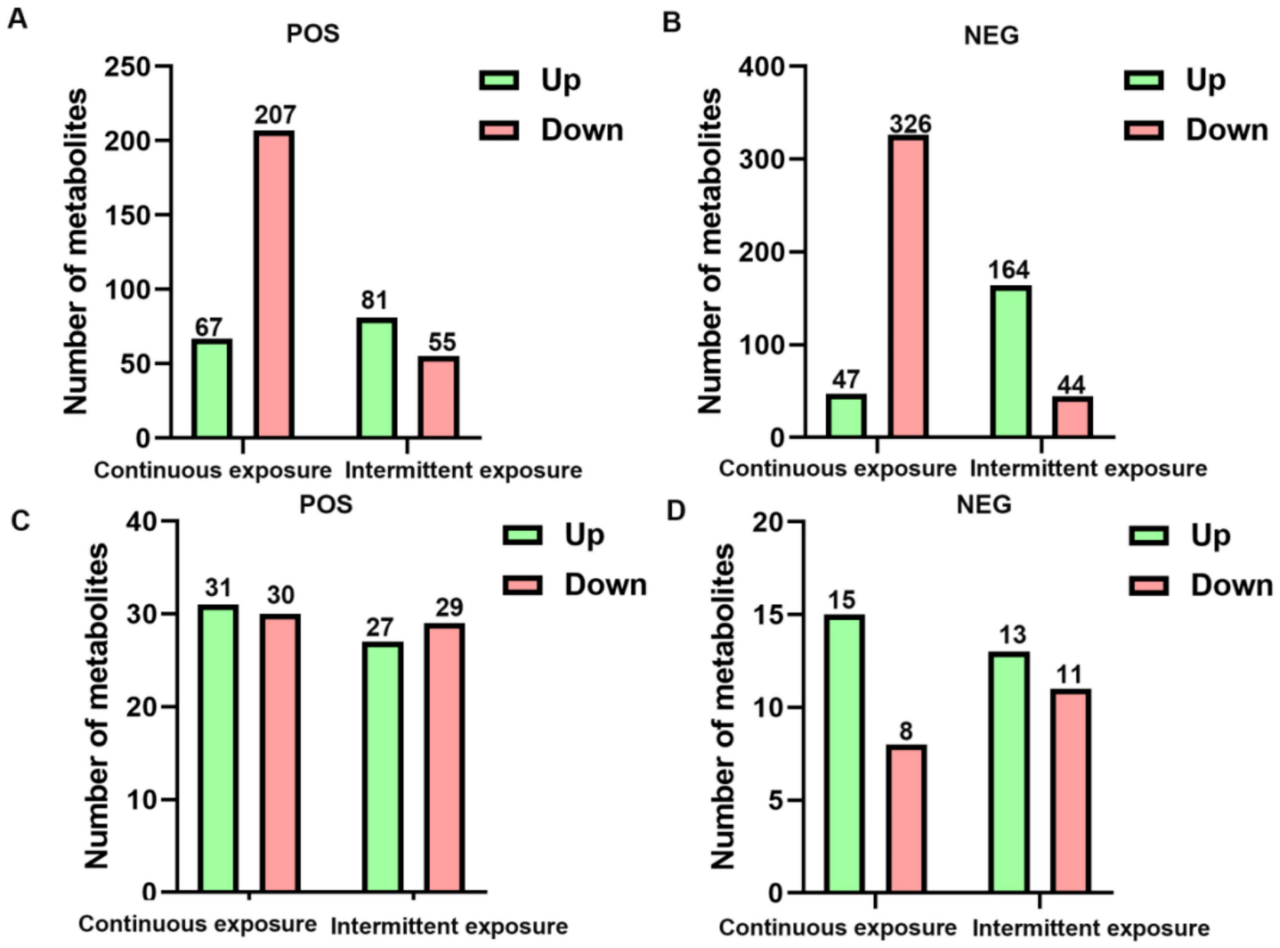
Differential metabolite expression in TM3 and GC-1 cells after different RF-EMF exposures. **(A)** Number of differential metabolites from the positive ion modes in TM3 cell. **(B)** Number of differential metabolites from the negative ion modes in TM3 cell. **(C)** Number of differential metabolites from the positive ion modes in GC-1 cell. **(D)** Number of differential metabolites from the negative ion modes in GC-1 cell.

We identified significantly altered metabolites in TM3 cells by combining VIP values from PLS-DA and *p*-values from univariate statistical analysis (*t*-test). In positive ion mode, continuous irradiation resulted in 274 differentially expressed metabolites (67 up-regulated and 207 down-regulated), while intermittent irradiation led to 136 differentially expressed metabolites (81 up-regulated and 55 down-regulated) (Figure 3A). In negative ion mode, continuous irradiation induced 373 differentially expressed metabolites (47 up-regulated and 326 down-regulated), whereas intermittent irradiation caused 208 differentially expressed metabolites (164 up-regulated and 44 down-regulated) (Figure 3B). These findings indicate that continuous irradiation had a more profound impact on TM3 cell metabolism compared to intermittent irradiation. In the GC-1 cell, in positive ion mode, continuous irradiation resulted in 61 differentially expressed metabolites (31 up-regulated and 30 down-regulated), while intermittent irradiation led to 56 differentially expressed metabolites (27 up-regulated and 29 down-regulated) (Figure 3C). In negative ion mode, continuous irradiation induced 23 differentially expressed metabolites (15 up-regulated and 8 down-regulated), whereas intermittent irradiation caused 24 differentially expressed metabolites (13 up-regulated and 11 down-regulated) (Figure 3D). Although the number of differentially expressed metabolites was lower than in TM3 cells, these changes still indicate that GC-1 cells respond to RF-EMF exposure, albeit to a lesser extent.

### The classification of differentially expressed metabolites in TM3 cells under different RF-EMF exposures

**Figure 4:**
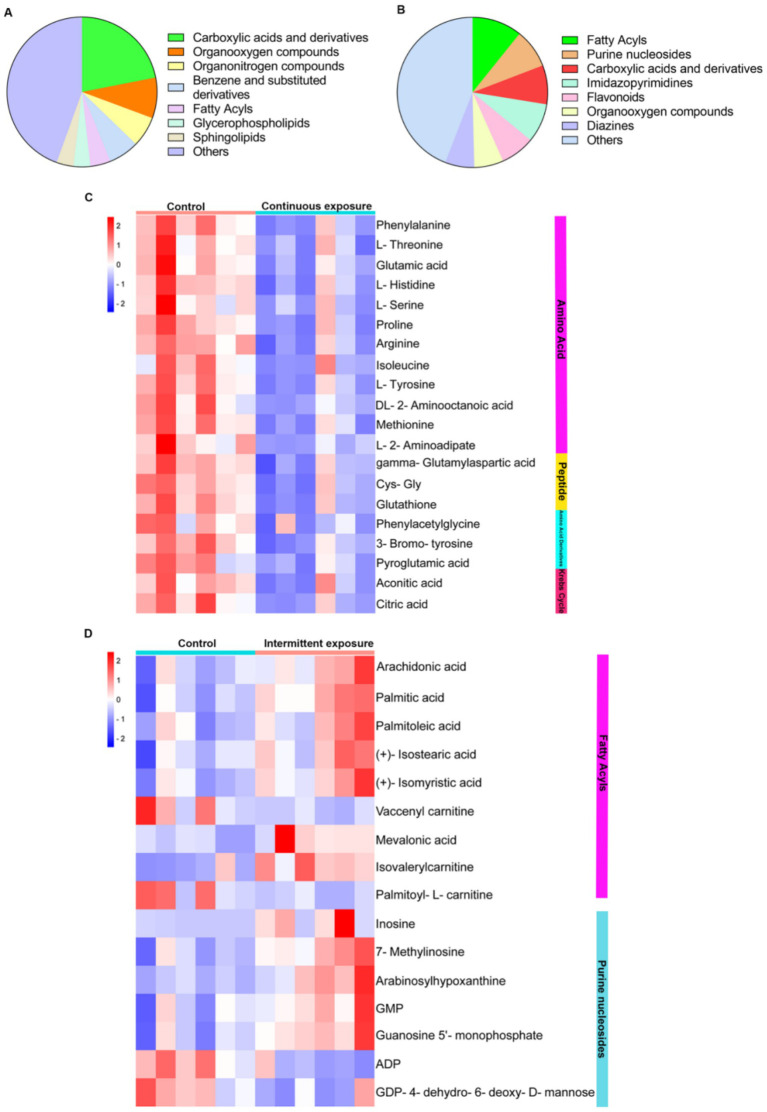
The classification of differential metabolites with different RF-EMFs radiation in TM3 cells. **(A)** The pie chart illustrates the distribution of different classes of differential metabolites induced by continuous RF-EMFs irradiation. **(B)** The pie chart illustrates the distribution of different classes of differential metabolites induced by intermittent RF-EMFs irradiation. **(C)** The heatmap of differential metabolites categorized into carboxylic acids and derivatives in TM3 cells under continuous RF-EMF exposure. **(D)** The heatmap of differential metabolites classified into fatty acyls and purine nucleosides under intermittent RF-EMFs irradiation.

We further conducted analysis on the classification of these differentially expressed metabolites, and the results are shown in Figures 4A,B. In the continuous irradiation, significant changes were observed in the levels of carboxylic acids and derivatives, organooxygen compounds, and organonitrogen compounds in TM3 cells. Among the carboxylic acids and derivatives, 12 amino acids, 3 peptides, 3 amino acid derivatives, and 2 organic acids exhibited the most notable alterations (Figure 4C). Specifically, the levels of amino acids such as phenylalanine, L-threonine, glutamic acid, L-serine, Arginine, proline and methionine were markedly reduced. The peptides with Cys-Gly, glutathione and gamma-Glutamylaspartic acid were also decreased significantly. Additionally, the levels of Aconitic Acid and Citric Acid also underwent significant decreases, indicating potential disruptions in the citric acid cycle following irradiation. The overall metabolic profile of TM3 cells post-irradiation suggests substantial perturbations, particularly in amino acid metabolism and the citric acid cycle, highlighting the sensitive nature of these biochemical pathways to RF-EMFs radiation-induced stress. We also carried out a detailed analysis of the categories of differential metabolites in TM3 cells subjected to intermittent irradiation. The findings clearly indicated that intermittent irradiation predominantly induced substantial alterations in the levels of fatty acyls and purine nucleosides within TM3 cells. Specifically, we noted remarkable elevations in arachidonic acid and palmitic acid post-irradiation, accompanied by significant increases in inosine, GMP (guanosine monophosphate), and guanosine 5′-monophosphate (Figure 4D). Conversely, there were notable decreases in ADP (adenosine diphosphate) and GDP-4-dehydro-6-deoxy-d-mannose (Figure 4D), indicating potential disruptions in energy metabolism and nucleotide pathways induced by intermittent exposure of RF-EMFs.

### Analysis of metabolic pathways in TM3 cells with different RF-EMFs irradiation

**Figure 5:**
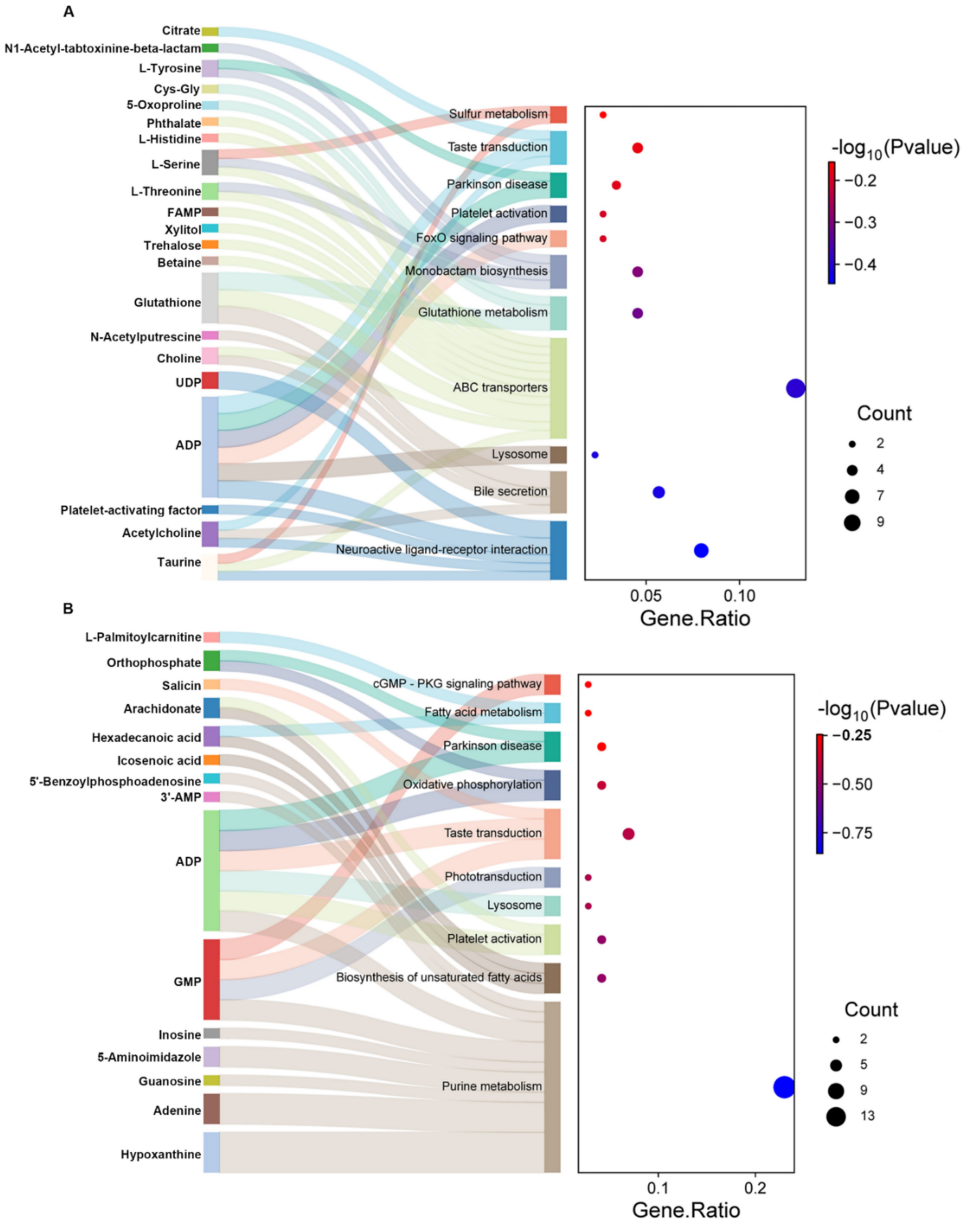
Analysis of metabolic pathways in TM3 cells with different RF-EMFs radiation. **(A)** KEGG Pathway analysis of differential metabolites induced by continuous RF-EMFs irradiation in TM3 cell (*p* < 0.05). **(B)** KEGG Pathway analysis of differential metabolites induced by intermittent RF-EMFs irradiation in TM3 cell (*p* < 0.05).

After identifying the differential metabolites among color morphs via pairwise contrasts, we performed KEGG enrichment to clarify metabolic pathways which were different among control group, continuous irradiation group and intermittent irradiation group. The top 10 enriched pathways in continuous irradiation group vs. control and intermittent irradiation group vs. control group, respectively, were shown in Figure 5. For the continuous irradiation treatment, KEGG analysis revealed that ABC transporters, Bile secretion, and glutathione metabolism were significantly enriched (Figure 5A). For the intermittent irradiation treatment, KEGG analysis revealed that purine metabolism, biosynthesis of unsaturated fatty acids and fatty acid metabolism were significantly enriched (Figure 5B). These results highlight the distinct metabolic responses induced by different irradiation modalities.

### Continuous exposure of RF-EMFs reduced the synthesis of glutathione in TM3 cell

**Figure 6:**
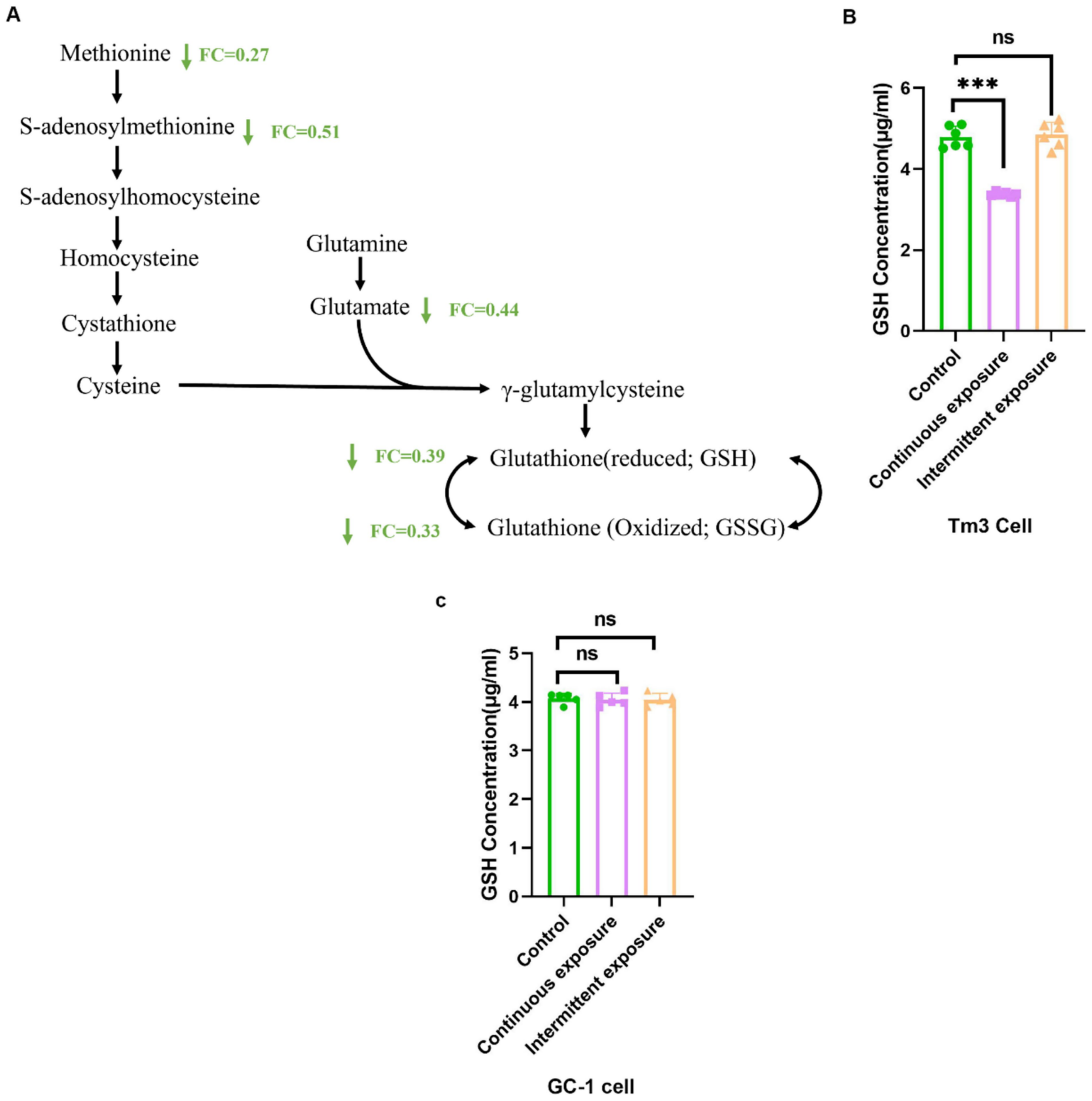
Continuous exposure of RF-EMFs reduced the level of glutathione in TM3 cell. **(A)** The metabolic pathway for the synthesis of glutathione from methionine. The metabolites highlighted in green are those that are down-regulated by exposure to RF-EMFs along this pathway. **(B)** The GSH level in TM3 cells after continuous or intermittent RF-EMF exposure. **(C)** The GSH level in GC-1 cells after continuous or intermittent RF-EMF exposure. The data are shown as means ± S. E. M. Statistical analyses were performed using one-way ANOVA followed by Tukey’s HSD *post hoc* test., with significance levels indicated as ***p < 0.001 (*N* = 6).

We conducted deeper data analysis on the glutathione metabolic pathway and observed that in TM3 cells, the concentrations of various metabolites along the pathway from methionine to glutathione decreased following irradiation, notably including methionine, S-adenosylmethionine, and glutamate (Figure 6A). Consequently, there was a substantial drop in both the reduced and oxidized forms of glutathione. Additionally, we employed ELISA experiments to quantify GSH levels in cells exposed to RF-EMFs. The results concurred, showing a significant decrease in GSH levels in TM3 cells upon continuous RF-EMF exposure, whereas intermittent exposure elicited no significant change (Figure 6B). By contrast, GC-1 cells exhibited no significant alteration in GSH content under either exposure pattern (Figure 6C). These findings imply that RF-EMF radiation may disrupt the normal metabolic processes involved in glutathione synthesis in a cell-type- and exposure-pattern-specific manner. Glutathione, a crucial antioxidant and detoxifying agent within cells, is pivotal in safeguarding against oxidative stress and maintaining cellular redox balance. The marked decrease in both reduced and oxidized glutathione levels observed exclusively in continuously exposed TM3 cells suggests a potential decrement in the cell’s capacity to counteract oxidative damage, which may heighten its vulnerability to cellular injury and dysfunction.

### Intermittent exposure of RF-EMFs promotes purine metabolism in TM3 cell

**Figure 7:**
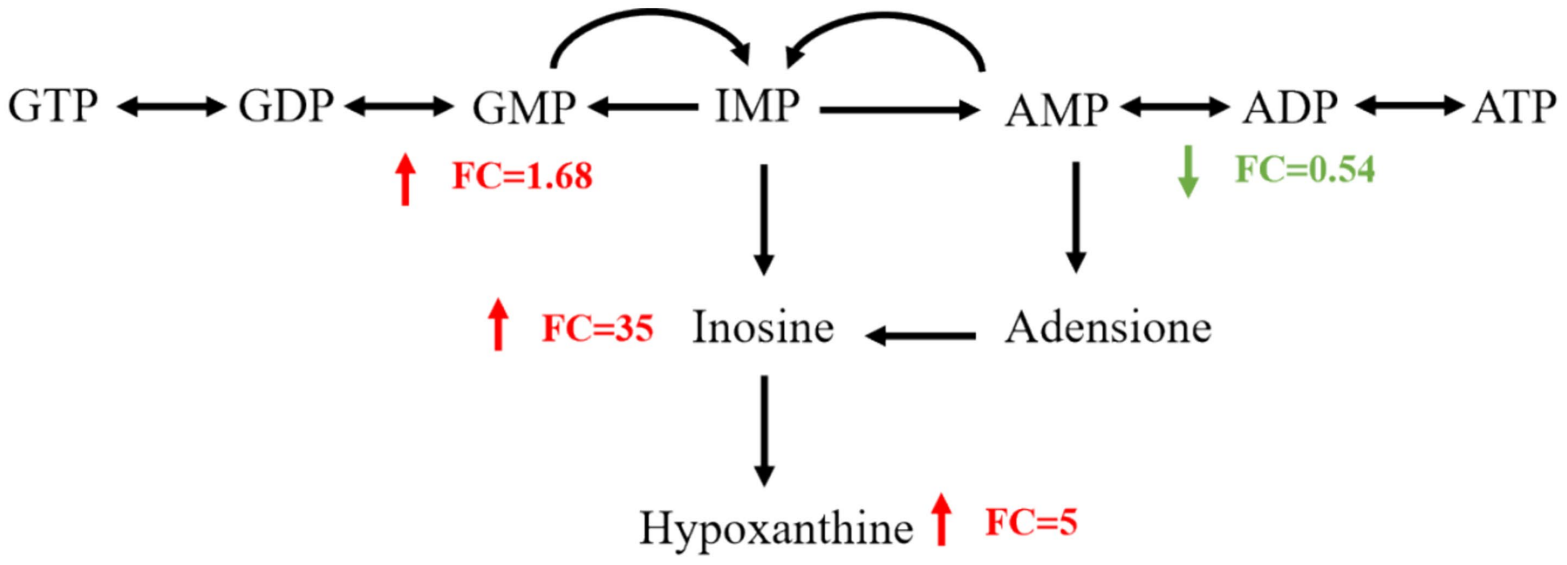
Intermittent exposure of RF-EMFs promotes purine metabolism in TM3 cell. The metabolic pathway for the purine metabolism. The green-highlighted metabolites are downregulated by RF-EMF exposure along this pathway, while the red-highlighted ones are upregulated.

We further conducted an in-depth analysis of the purine metabolism pathways induced by intermittent RF-EMFs radiation. The results, as illustrated in the Figure 7, indicate that intermittent RF-EMFs radiation causes a significant decrease in ADP levels, while inosine and hypoxanthine, which are products of nucleotide degradation, are upregulated following irradiation. These results showed that intermittent RF-EMF exposure disrupts purine metabolism, highlighting potential impacts on cellular energy regulation and homeostasis.

### The shared pathways altered in both continuous and intermittent exposure of RF-EMFs in TM3 cells

**Figure 8:**
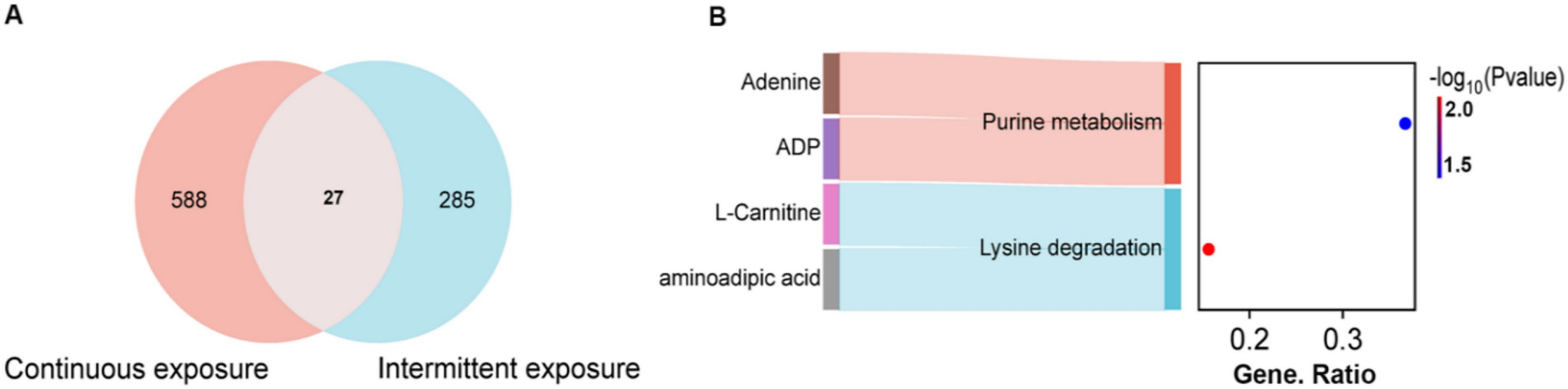
The pathways shared in continuous and intermittent exposure of RF-EMFs in TM3 cell. **(A)** The Veen analysis of the common differential metabolites induced by continuous and intermittent RF-EMFs irradiation in TM3 cell. **(B)** The KEGG Pathway analysis of the common differential metabolites induced by continuous and intermittent RF-EMFs irradiation in TM3 cell (*p* < 0.05).

We further conducted Venn analysis on the differential metabolites in TM3 cells after continuous and intermittent irradiation to find the common pathways induced by RF-EMFs irradiation. As illustrated in Figure 8A, among the differential metabolites identified in these two groups, a total of 27 metabolites were altered under both irradiation conditions. Subsequently, we performed KEGG enrichment analysis on these 27 metabolites, revealing two pathways with *p*-values less than 0.05: purine metabolism and lysine degradation (Figure 8B). Notably, ADP, adenine, L-carnitine, and aminoadipic acid emerged as key molecules involved in the cellular metabolic alterations caused by RF-EMF irradiation.

### Metabolomics-proteomics network analysis in TM3 cells with different RF-EMFs radiation

**Figure 9:**
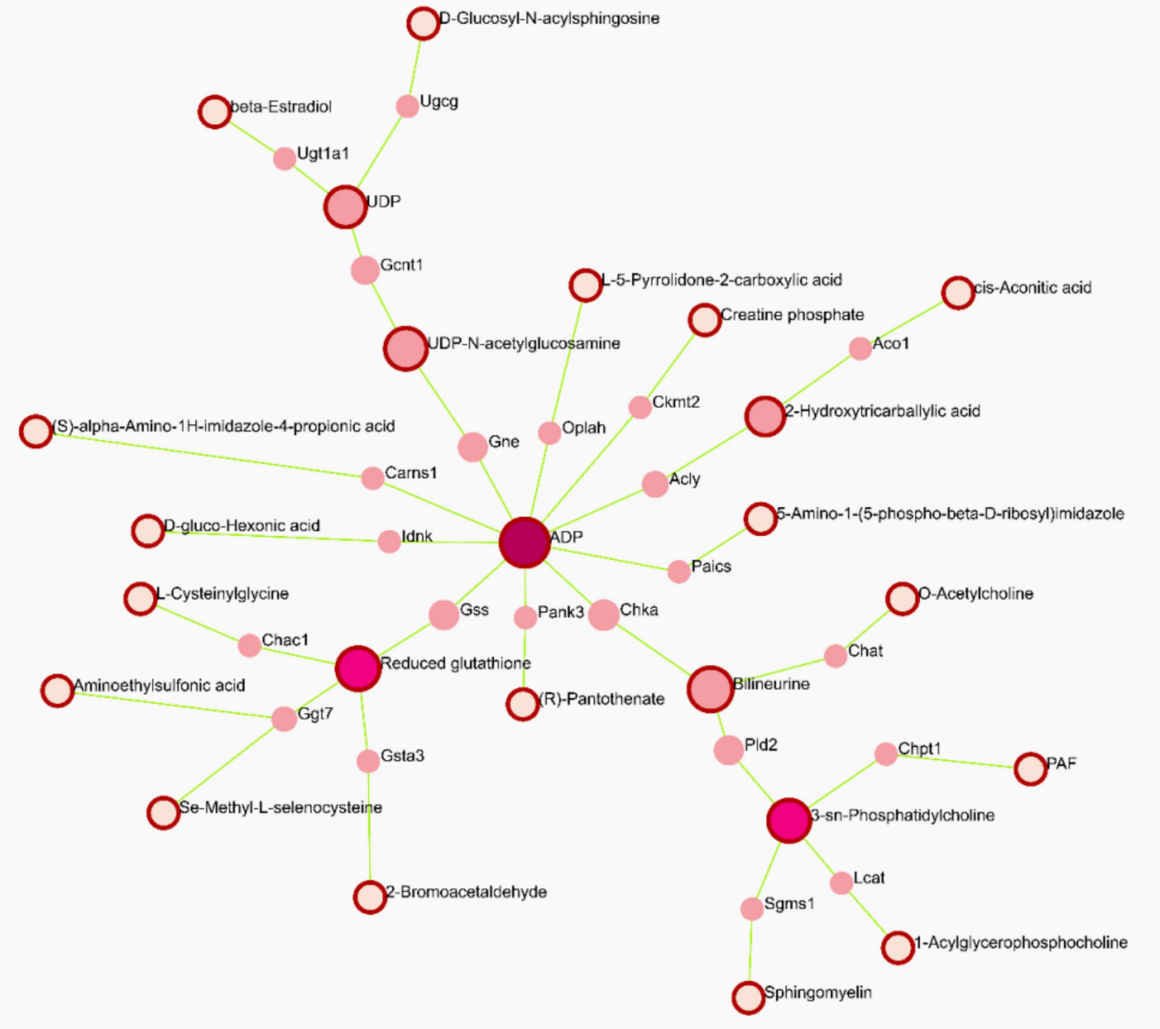
Analysis of differentially expressed metabolites and target genes by continuous RF-EMFs irradiation in TM3 cell. Constructing Analysis of differentially expressed metabolites and target genes based on the OmicsNet website. The notes with outer frames represent the differential metabolites induced by continuous RF-EMFs irradiation in TM3 cell, while the notes without outer frames represent target genes (*p* < 0.05).

**Figure 10:**
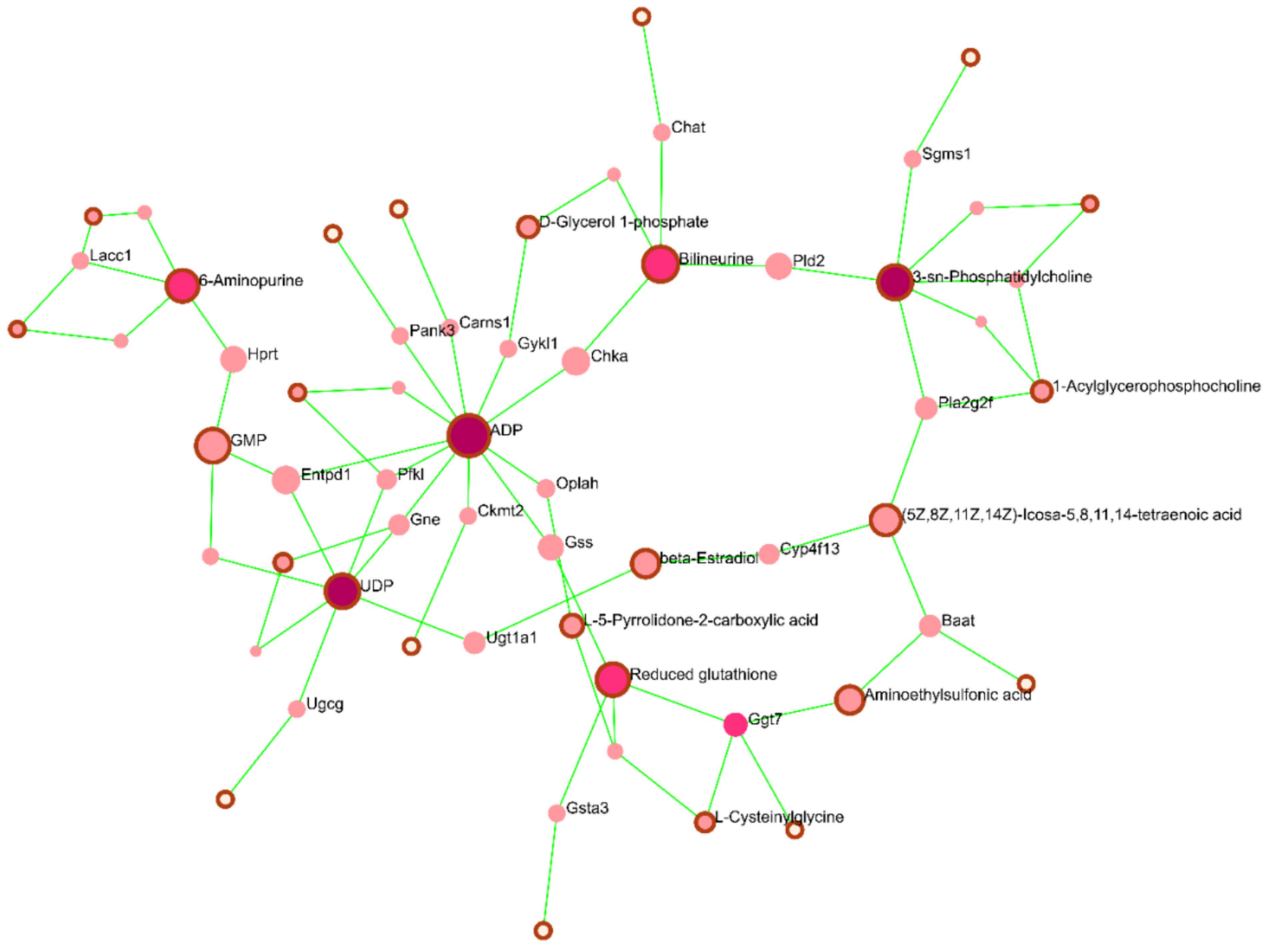
Analysis of differentially expressed metabolites and target genes in intermittent irradiation in RF-EMFs TM3 cell. Constructing Analysis of differential expressed metabolites and target genes based on the OmicsNet website. The notes with outer frames represent the differential metabolites induced by intermittent RF-EMFs irradiation in TM3 cell, while the notes without outer frames represent target genes (*p* < 0.05).

To further explore regulatory relationships of the biologically related molecules, we constructed a metabolites-proteomics network of differential metabolites and regulatory target genes based on the OmicsNet website, the results of which were represented by an integrated network. As shown in Figure 9, we can observe the situation regarding the differential metabolites induced by continuous RF-EMFs irradiation and the genes that may be closely related to the changes in these metabolites. We can see that the changes in ADP caused by continuous RF-EMFs irradiation may occupy a crucial position among the overall metabolic changes. The alterations in the activity of related proteins triggered by changes in ADP levels may have influenced the variations of other metabolites, such as glutathione and UDP. Similarly, intermittent irradiation yields the similar results (Figure 10). Therefore, these findings highlight the central role of ADP in mediating the metabolic response to RF-EMF exposure.

### Differential metabolite expression in GC-1 cells after RF-EMFs irradiation

**Figure 11:**
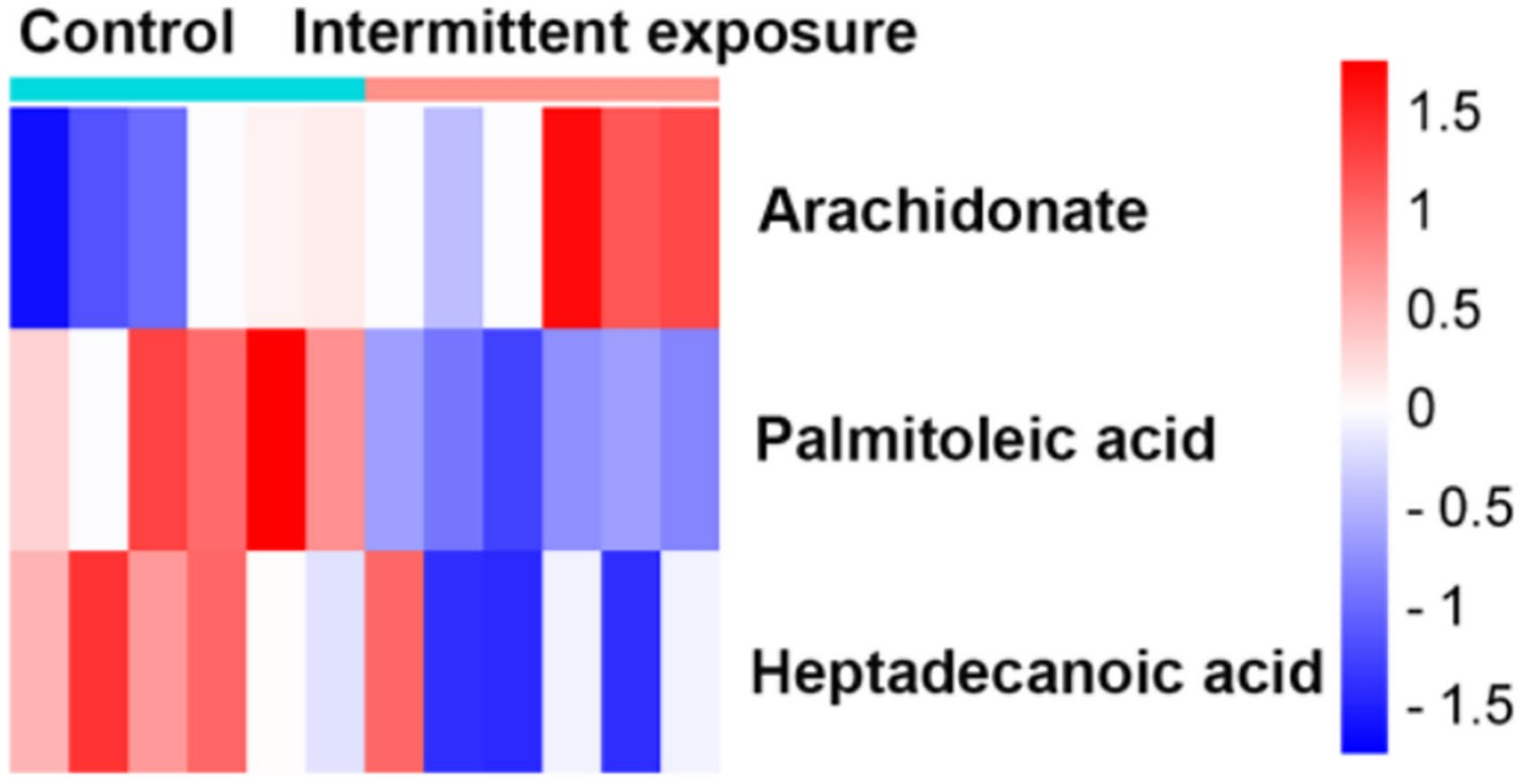
Intermittent exposure of RF-EMFs influenced the lipid metabolism in GC-1 cell. The heatmap of metabolism of Palmitoleic acid, Arachidonate, and Heptadecanoic acid.

The KEGG analysis were also performed to clarify metabolic pathways for the GC-1 cells, unfortunately, for the continuous irradiation treatment, no significantly enriched metabolic pathways were identified using the screening criterion of *p* < 0.05. In contrast, when comparing the intermittent irradiation group to the control group, only the fatty acid biosynthesis pathway involving palmitoleic acid was found to be enriched. A comprehensive analysis of the differential metabolites in GC-1 cells after intermittent irradiation revealed that three of these metabolites are associated with lipid metabolism: palmitoleic acid, arachidonate, and heptadecanoic acid (Figure 11). Specifically, arachidonate levels were upregulated following irradiation, whereas the levels of palmitoleic acid and heptadecanoic acid were significantly downregulated. These findings suggest that intermittent irradiation has a specific impact on the lipid metabolism profile of GC-1 cells, resulting in significant alterations in the levels of key fatty acids involved.

## Discussion

In this study, we conducted a comprehensive metabolomic analysis to investigate the impact of RF-EMFs on the metabolism of TM3 and GC-1 cells. The results provide valuable insights into the potential mechanisms underlying the effects of RF-EMFs on reproductive health.

Our study revealed significant metabolic alterations in TM3 cells following 1950MHZ RF-EMF continuous exposure. The changes in carboxylic acids and derivatives, including amino acids and the citric acid cycle, are particularly noteworthy. Amino acids are not only building blocks for proteins but also play essential roles in energy metabolism and cell signaling (21). The decreased levels of specific amino acids such as phenylalanine, L-threonine, glutamic acid, proline, and methionine, along with the reduction in glutathione and related peptides, suggest a disruption in normal cellular processes. It is well known that GSH depletion can exacerbate oxidative stress, thereby reducing NADPH bioavailability; this NADPH deficit, in turn, disrupts the homeostasis of fatty-acid synthesis and mitochondrial function (22, 23). Some studies indicate that specific RF-EMF regimens do not disturb glutathione homeostasis; for instance, HL-60 cells exposed to 2.45 GHz RF-EMF (217 Hz pulse, 0.1 W/kg SAR) showed no changes in GSH content (24). However, a larger body of evidence demonstrates that RF-EMFs exposure can affect GSH levels in various cell types (3, 25, 26), and our findings showed that 1950MHZ continuous exposure reduced the GSH level in TM3 cell, which is align with this. But also, intermittent exposure left GSH unchanged in TM3 cell and in GC-1 cell, in line with previous reports that only certain exposure paradigms perturb glutathione balance. Collectively, these data confirm that the impact of 1950 MHz RF-EMF on GSH is both cell-type- and exposure-pattern-specific.

We also observed a notable decline in the concentrations of key precursors involved in glutathione synthesis, including methionine, S-adenosylmethionine, and glutamate, following RF-EMF exposure. This indicates that, in addition to reducing glutathione synthesis by decreasing the activity of enzymes involved in glutathione synthesis (27), the decreased levels of its amino acid precursor substrates play a crucial role in the subsequent reduction of glutathione levels after RF-EMF exposure. The reduced GSH levels could potentially impair the cell’s ability to protect against oxidative damage, which is especially critical for reproductive cells as they are highly sensitive to oxidative stress (28). This may in turn affect sperm production and quality, as Leydig cells are involved in testosterone secretion, which is essential for spermatogenesis.

The enrichment of ABC transporters and bile secretion pathways in TM3 cells after 1950 MHz continuous irradiation implies that the cell’s ability to transport and detoxify substances may be compromised. This could lead to the accumulation of harmful metabolites and further disrupt cellular homeostasis. No previous study has reported RF-EMF–induced alterations in pathways involving ABC transporters and bile secretion; thus, the present findings lay the groundwork for further investigations into their role under RF-EMF exposure. The alterations in fatty acyls and purine nucleosides following intermittent irradiation suggest potential disruptions in energy metabolism and nucleotide pathways. Given the high energy demands of spermatogenesis (29), any impairment in energy metabolism could have a negative impact on sperm development.

In GC-1 cells, the intermittent irradiation-induced changes in lipid metabolism, specifically the alterations in palmitoleic acid, arachidonate, and heptadecanoic acid levels, may affect membrane fluidity and integrity (30). The plasma membrane of spermatogonia cells is crucial for maintaining their normal function and interaction with the surrounding environment. Changes in lipid composition could disrupt these processes and potentially affect spermatogonial stem cell self-renewal and differentiation, which are essential for the continuous production of sperm.

The identification of shared pathways between continuous and intermittent irradiations, such as purine metabolism and lysine degradation, provides further evidence of the potential impact of RF-EMFs on reproductive cells. Purine metabolism is involved in nucleotide synthesis and energy transfer, and disruptions in this pathway could affect DNA replication and repair, as well as cellular energy balance (31). Lysine degradation is also an important metabolic process, and alterations in this pathway may have implications for protein synthesis and modification (32).

Under both continuous and intermittent RF-EMF exposures in TM3 cell, intracellular ADP exhibited a consistent downward trend, suggesting that ADP may function as a pivotal metabolic node. The consequent changes in the ATP/ADP ratio could influence AMPK phosphorylation, thereby decelerating the tricarboxylic acid cycle (evidenced by reductions in citrate, *α*-ketoglutarate and malate) and impairing glutathione synthesis (33, 34). Such alterations potentially link mitochondrial energy homeostasis to a decline in steroidogenic capacity within TM3 cells.

Recent evidence indicates that 900 MHz RF-EMF from mobile phones can compromise testicular immunity and elevate the risk of germ-cell tumors (14). Although our 1950 MHz study did not assess oncogenic endpoints, the observed glutathione depletion, purine imbalance and membrane-lipid remodeling align mechanistically with early carcinogenic signatures reported at 900 MHz. These convergent findings underscore the need for dose–response and multi-frequency evaluations to clarify whether metabolic perturbations precede malignant transformation across different RF carriers.

The differential sensitivity of TM3 and GC-1 cells to RF-EMF irradiation highlights the importance of cell type-specific responses. TM3 cells, which are involved in testosterone production and have higher metabolic activity, exhibited significant alterations in amino acid metabolism, the citric acid cycle, and glutathione synthesis. In contrast, GC-1 cells, which are primarily involved in spermatogonial stem cell maintenance, showed limited metabolic changes, with only intermittent irradiation affecting fatty acid biosynthesis. These findings suggest that the metabolic impact of RF-EMF exposure may be closely related to the functional roles and metabolic demands of different cell types within the testis.

Overall, our results suggest that RF-EMFs can induce significant metabolic changes in reproductive cells, which may ultimately affect their function and contribute to the potential risks associated with RF-EMF exposure on reproductive health. However, further studies are needed to fully understand the complex mechanisms involved and to determine the long-term consequences of these metabolic alterations. Additionally, more research is required to explore potential preventive or protective measures to safeguard reproductive health in the context of increasing RF-EMF exposure.

## Conclusion

Our results showed that continuous irradiation had a more pronounced impact on the metabolism of TM3 cells, primarily affecting amino acid metabolism, the citric acid cycle, ABC transporters, bile secretion, and glutathione metabolism. In contrast, intermittent irradiation mainly altered the levels of fatty acyls and purine nucleosides, with significant enrichment in purine metabolism, biosynthesis of unsaturated fatty acids, and fatty acid metabolism. Compared to TM3 cells, GC-1 cells exhibited lower sensitivity to RF-EMF irradiation. Both irradiation modalities affected purine metabolism and lysine degradation pathways in TM3 cells, suggesting that changes in ADP levels may serve as a key metabolic signature in the cellular response to RF-EMF exposure.

### Limitations

This study revealed that 1950 MHz radiofrequency electromagnetic fields, applied under different exposure patterns, can modulate the metabolic profiles of GC-1 and TM3 cells. Nevertheless, these findings derive solely from an *in vitro* cell-based system. Given the complexity of the *in vivo* reproductive milieu-encompassing hormonal regulation, immune responses, and intercellular signaling- cellular models cannot fully recapitulate these interactions. Follow-up investigations in whole-animal models are therefore planned to corroborate and extend the present observations. Another limitation of this study is that only one frequency and one SAR level were employed; although continuous and intermittent exposure patterns were compared, the resulting frequency-dose–response landscape remains incomplete. Future investigations should therefore adopt a multi-frequency, multi-dose design to provide a comprehensive characterization of these relationships.

## Data Availability

The raw data supporting the conclusions of this article will be made available by the authors, without undue reservation.

## Footnotes

^1^ http://www.genome.jp/kegg

^2^ https://www.omicsnet.ca/
